# IL-15/IL-15Rα Heterodimeric Complex as Cancer Immunotherapy in Murine Breast Cancer Models

**DOI:** 10.3389/fimmu.2020.614667

**Published:** 2021-02-08

**Authors:** Siqi Guo, Ronald B. Smeltz, Anthony Nanajian, Richard Heller

**Affiliations:** ^1^ Frank Reidy Research Center for Bioelectrics, Old Dominion University, Norfolk, VA, United States; ^2^ Department of Microbiology & Immunology, Virginia Commonwealth University, Richmond, VA, United States; ^3^ Department of Medical Engineering, University of South Florida, Tampa, FL, United States

**Keywords:** breast cancer, interleukin-15/interleukin-15 receptor alpha complex, cancer immunotherapy, gene electrotransfer, depletion of myeloid derived suppressor cells

## Abstract

Interleukin 15 (IL-15) has been evaluated as a potential treatment for solid tumors in clinical trials, but the effectiveness of systemic IL-15 administration as a monotherapy has not been realized. IL-15 receptor alpha (IL-15Rα) can stabilize IL-15 and enhance its bioactivity. The goal of this study was to examine the activity of IL-15/IL-15Rα complex (IL-15cx) to CD8^+^ T cells and evaluate its potential efficacy in murine breast cancer models. The antitumor efficacy was studied in mouse mammary carcinoma models (Her2/neu transgenic and 4T1-luc mammary cancers) treated with systemic recombinant protein with/without the depletion of myeloid-derived suppressor cells or intra-tumoral gene electrotransfer (GET). IL-15cx shows superior *in vivo* bioactivity to expand CD8 T cells in comparison to an equimolar single chain IL-15. T-bet is partially involved in CD8 T cell expansion *ex vivo* and *in vivo* due to IL-15 or IL-15cx. Intraperitoneal administration of IL-15cx results in a moderate inhibition of breast cancer growth that is associated with an increase in the frequency of cytotoxic CD8 T cells and the improvement of their function. The depletion of myeloid-derived suppressor cells (MDSCs) has no impact on mouse breast cancer growth. IL-15cx treatment diminishes MDSCs in murine tumors. However, it also antagonizes the effects of anti-Gr-1 depleting antibodies. Intratumoral GET with plasmid IL-15/IL-15Rα leads to a long-term survival benefit in 4T1 mammary carcinoma model. An early increase of local cytotoxic cells correlates with GET treatment and an increase of long-term memory T cells results from animals with complete tumor regression. Systemic and local administration of IL-15cx shows two distinct therapeutic responses, a moderate tumor growth inhibition or heterogeneous tumor regressions with survival improvement. Further studies are warranted to improve the efficacy of IL-15cx as an immunotherapy for breast cancer.

## Introduction

A major breakthrough in cancer therapy is immune checkpoint inhibitor (CPI)-based immunotherapy which has been approved by the FDA for the treatment of multiple types of cancers (1). However, some types of cancer are largely resistant to this powerful immunotherapy. Resistance is typically related to the immunogenicity of the tumor. One of these poorly immunogenic tumors is breast cancer with response rates of only 0%–18.5% to CPI therapy alone (2–4). Atezolizumab, an anti-PD-L1 antibody is the first checkpoint inhibitor approved by FDA in combination with chemotherapy to treat triple-negative and PD-L1 positive metastatic breast cancer. Triple negative breast cancer only accounts for approximately 20% of all breast cancer cases. PD-L1 is expressed mainly on tumor-infiltrating immune cells in roughly 41% of patients with triple negative breast cancer (5). Considering these two percentages together, there are only a small subset of breast cancer patients who would benefit from this combination therapy. Other immunotherapeutic strategies and combination approaches are needed to improve the therapeutic efficacy and broaden its indications for a large patient population.

Interleukin 15 (IL-15) is a pleiotropic cytokine, which was originally discovered by Drs. Waldmann and Grabstein’s groups (6, 7). IL-15 is considered superior to IL-2 for cancer immunotherapy due to its strong bioactivity in improving CD8^+^ T and natural killer (NK) cell survival, proliferation and cytotoxic function (8). It also delivers its function without activating regulatory T cells (Tregs) (9) and mediating activation-induced cell death of effector cytotoxic T cells (10). IL-15 has been studied in clinical trials as a potential treatment for various solid tumors; however, its systemic administration has not been demonstrated to be an effective monotherapy (11–13). In its monomeric form, IL-15 has a very short half-life requiring continuous intravenous infusion (14). A significant discovery in the IL-15 immunobiological research is the trans-presentation of IL-15 *via* pre-associated IL-15/IL-15 receptor alpha (IL-15Rα) (15–17). By taking advantage of this mechanism, several IL-15 superagonist fusion proteins and IL-15/IL-15Rα complex (IL-15cx) have been generated and studied in animal models and clinical trials (18). These IL-15 superagonists exhibit prolonged half-lives and an increased potent bioactivity to T cells and NK cells. IL-15cx or IL-15cx trans-presenting cells show superior bioactivity and antitumor efficacy to IL-15 monomer in multiple preclinical tumor models (18–20). IL-15cx has been reported to effectively treat murine melanoma and pancreatic tumors (21–23). There are no studies on the therapeutic efficacy of IL-15cx treatment for breast cancer whereas an IL-15 superagonist mutant (IL-15N72D) and dimeric IL-15RαSushi-Fc fusion protein or N803 has been reported to prolong the survival of breast cancer bearing mice with significant antimetastatic activity but no impact on primary tumor growth (24, 25).

In this study, we examined the bioactivity of IL-15cx to CD8 T cells and explored whether T-bet was involved in IL-15cx driving CD8 T cell expansion. Antitumor activity of IL-15 was evaluated in two murine breast cancer models, mouse Her2/neu^+^ and 4T1-luc mammary cancers treated with systemic administration and intratumor delivery of plasmid IL-15/IL-15Rα, respectively. The potential efficacy of combination therapy with depletion of myeloid-derived suppressor cells (MDSCs) was studied as well. In addition, immune cells responding to IL-15cx therapy were identified to help understand the immunobiology of antitumor activity

## Materials and Methods

### Tumor Cell Lines

Mouse Her2/neu^+^ mammary carcinoma (MMC) was obtained from Dr. Manjili ‘s group at Virginia Commonwealth University who established MMC cell lines from the spontaneous mammary tumor of Her2/neu transgenic FVBN202 mice (26). 4T1-luc murine mammary cancer cells were originally provided by Dr. Gary Sahagian at Tufts University (27) and maintained in high-glucose Dulbecco’s Modified Eagle Medium (ATCC^®^ 30–2002™) (ATCC)-supplemented with 10% fetal bovine serum (FBS), non-essential amino acids, and antibiotics (100 units/ml penicillin and 100 μg/ml streptomycin) (three items above from Atlanta Biologicals).

### Reagents and Antibodies

Mouse CD4^+^CD25^+^ T_reg_ isolation kit (Cat#130-091-041), Anti-thy 1.2 Microbeads (Cat# 130-091-376), mouse CD8α+ T cell isolation kit (Cat# 130-104-075) (Miltenyi Biotech); recombinant human IL-15 (Cat#247-ILB-025), recombinant mouse IL-15Rα/Fc chimeric protein (Cat#551-MR-100), anti-Gr-1 monoclonal Ab (Cat#MAB1037-500) (R&D); Carboxyfluorescein succinimidyl ester (CFSE) (Cat#87444-5MG), 5 mM in DMSO (Sigma); low-endotoxin, azide-free anti-CD3 antibody (2C11 clone), anti-CD16/32 (Fc block) (Cat#101319), rat anti-mouse CD8 APC Cy7 (Cat# 100714, RRID : *AB_312753*)), rat anti-mouse CD4 FITC (Cat# 100406, RRID : *AB_312691*), rat anti-mouse Gr-1 APC (Cat# 108412, *RRID : AB_313377*), and rat anti-mouse CD11b PE (Cat# 101208, *RRID : AB_31279*1), hamster anti-mouse CXCR3 PE (Cat# 126505, *RRID : AB_1027656*), rat anti-mouse CD44 APC (Cat# 103012, *RRID : AB_312963*), rat anti-mouse CD62L PE (Cat# 104408, *RRID : AB_31309*), hamster anti-mouse CD103 PE (Cat# 121406, *RRID : AB_1133989*), rat anti-mouse CD3 PE (Cat# 100206, RRID : *AB_312663*) and rat anti-mouse NKp46 PE Cy7 (Cat# 137603, *RRID : AB_10552741*) (Biolegend); mouse IFN-γ ELISA Set (Cat#555138) (BD biosciences) were obtained from indicated vendors, respectively. Plasmids IL-15/IL-15Rα expressing mouse IL-15 and IL-15Rα was provided by Drs. B. Felber and G. Pavlakis (National Cancer Institute, Bethesda, MD) and commercially produced by Aldevron, (Fargo, ND).

### Mice

C57BL/6, T bet knock-out B6.129S6-*Tbx21^tm1Glm^* (T-bet^-^), female Balb/c and parental FVB mice were purchased from Jackson Laboratories ((Bar Harbor, ME)). Female Her2/neu transgenic FVBN202 mice were obtained from Charles River Laboratories (Wilmington, MA). FVBN202 mice over-express a non-activated rat *neu* transgene under the regulation of the mouse mammary tumor virus promoter (28). T-bet^-^ mice are lack of T-box transcription factor, TBX21 (29). C57BL/6 and T-bet^-^ mice were used to study *ex vivo* and *in vivo* proliferation of cytotoxic CD8 T cells. Female FVB and FVB202 mice were used to establish MMC tumor model whereas female Balb/c mice were utilized to establish 4T1-luc orthotopic breast tumor model.

### *Ex Vivo* Proliferation of Cytotoxic T Cells

CD8 T cells isolated from C57BL/6 or T-bet mice and with purity over 95% (**Supplementary Figure 1**) were labeled with CFSE (5 µM) (30). The IL-15/IL-15Rα-Fc complex was prepared by the incubation of IL-15 plus IL-15Rα-Fc in a ratio 1: 6 (by weight) in PBS at 37°C for 30 min. Of note, the excess amount of IL-15Rα-Fc was used here to block any free IL-15 in the media. CFSE labeled CD8 T cells (2 x 10^5^) were placed into a well of a 24-well plate with no cytokine (control), IL-15 (1, 5 or 10 ng/ml), IL-15Rα-Fc (6, 30, 60 ng/ml) or IL-15/IL-15Rα-Fc complex (IL-15cx) (7, 35 or 70 ng/ml containing IL-15 and IL-15Rα-Fc at respective concentration) and incubated at 37°C with 5% CO_2_ for 5 days. Cells were harvested to analyze the proliferation of T cells by flow cytometer (Beckman-Coulter FC500). Quantification of proliferation was determined by gating on both diluted CFSE^+^ cells and CD8^+^ T cells and determining the frequency of cells in a total live cell gate.

### The Suppression of CTL Proliferation by Tregs

A 48-well plate was precoated with low-endotoxin/azide-free anti-CD3 antibody at 0.5 *μ*g/ml for overnight. Splenocytes depleted of T cells from C57BL/6 were used as antigen presenting cells (APC) after exposure to 10 Gy of radiation. Natural CD4^+^CD25^+^ Tregs were isolated from lymph nodes. 2.5 × 10^5^ CFSE-labeled CD8 T cells, 1 × 10^6^ APC and variable numbers of Tregs with or without IL-15 1 ng/ml were cocultured in a 48-well plate. 3 days later, cells were stained with anti-CD8 Ab and analyzed by flow cytometer (Beckman-Coulter FC500). Treg suppressor function was calculated: *Suppressor function (%) = [(% dividing CD8^+^ T cells without Tregs* − *% dividing CD8^+^ T cells with Tregs)/% dividing CD8^+^ T cells without Tregs] x 100*.

Supernatants from this assay were collected and stored at -80°C for further analysis of IFN-*γ by ELISA.*

### *In Vivo* Proliferation of Cytotoxic T Cells

CFSE labeled CD8 T cells (5 x 10^6^ in 100 µl RPMI) isolated from C57BL/6 or T-bet^-^ mice were administrated intravenously *via* tail vein into mice. On the same day, IL-15 (2.5 µg) or preformed IL-15cx (12.5 µg containing 2.5 µg IL-15 and 10 µg IL-15Rα-Fc) was given intraperitoneally (i.p.) into mice. Five days later, mice were euthanized. Spleens and lymph nodes were harvested to assess the proliferation and CXCR3 expression of T cells.

### MMC Tumor Model and Systemic Cytokine Treatment With or Without Depletion of MDSCs

Female FVB parent or FVBN202 transgenic mice at 6–10 weeks of age were injected in the left posterior mammary fat pad with 5 × 10^6^ neu^+^-MMC cells. After tumor inoculation days 5 and 10, animals were treated with i.p. PBS (as controls), IL-15 (2.5 µg), IL-15Rα-Fc (10 µg), or IL-15cx complex (12.5 µg) of which two components were mixed as the same molar ratio and incubated 30 min at 37°C before treatment. To deplete MDSCs, anti-Gr-1 Ab (100 µg) was given i.p. to the tumor bearing animals after tumor inoculation days 4, 9, and 14. Tumor sizes were measured twice a week by a digital caliper. The calculation of tumor volume was described in our previous publication (31). The studies have been reviewed and approved by the Institutional Animal Care and Use Committee (IACUC) at Virginia Commonwealth University.

### 4T1-luc Breast Tumor Model and Intra-Tumoral Gene Electrotransfer (GET)

A pilot study demonstrated that mice immunized with frozen/thawed 4T1-luc cells resulted in no protection, thus luciferase transgenic 4T1 cells (4T1-luc) are not immunogenic (manuscript under preparation). 1 × 10^6^ 4T1-luc cells were inoculated in the left posterior mammary fat pad of female Balb/c mice aged 8-10 weeks. Tumor sizes were measured twice a week by a digital caliper. When tumor sizes reached 4 to 6 mm (equivalent to 30 ~ 80 mm^3^), mice were anesthetized and intra-tumoral GET was performed. The DNA plasmid pIL-15/IL-15Rα (50 µl at 2 µg/ml) was administered directly into tumors then electric pulses were applied by a two-plate electrode using a BTX ECM 830 pulse generator. Pulse parameters were 5 ms, 600 V/cm, 10 pulses and 1 Hz (32). GET was performed three times at days 0, 4 and 7. The study has been reviewed and approved by the IACUC at Old Dominion University.

### IFN-γ Release From Splenocytes to Antigen Recall and ELISA

Splenocytes (2 × 10^5^), which were prepared from parent FVB or FVBN202 transgenic mice with various treatments, were incubated in a 96-well cell culture plate at 37°C for 24 h with medium alone (control), irradiated neu^-^ MMC or neu^+^ MMC (2 × 10^4^), respectively. Supernatants were collected to quantify IFN-γ release by ELISA following the manufacturer instructions. A standard curve was made by six two-fold serial dilutions of the mouse IFN-γ protein (the range of concentration between 15.6–1,000 pg/ml). The IFN-γ concentrations of samples were calculated from the standard curve. Samples were re-evaluated with appropriate dilutions if the concentrations of samples were out of the range of standard curve.

### Flow Cytometry

All cells from *ex vivo* cell culture experiments, or 1 to 2 million peripheral blood mononuclear cells (PBMCs), splenocytes or lymphocytes were collected and suspended in 100 µl complete media or FACS buffer (2% FBS in DPBS), then cells were added to a single antibody or antibody mixture and incubated at 4°C for 45 min. Cells were washed with 2 ml FACS buffer twice and re-suspended in 0.5 ml FACS buffer with 2.5% paraformaltehyde for flow cytometric analysis by FACSAria (BD Biosciences) or MACSQuant^®^ Analyzer 10 (Miltenyi Biotec). To analyze effector/memory T cells in blood, spleen and lymph nodes, CD3^+^CD4^+^ or CD3^+^CD8^+^ cells were gated for the analysis of CD44 and CD62L expression on these double positive cells.

### Statistical Analysis

All values from experimental groups were reported as the mean ± standard deviation (SD) or standard error (SE) indicated in Figure legends, respectively. Animals were grouped randomly based on tumor sizes before treatments. Experiments in this study generated quantitative changes of immune cells and their function including CD8^+^ T cells, CXCR3^+^ CD8^+^ T cells, CD4^+^ T cells, CD44^+^CD62^+^/CD44^+^CD62^-^ central/effector memory T cells, CD8^+^CD103^+^ tissue resident memory T cells, CD3^+^NKp46^+^ NKT cells, CD11b^+^Gr-1^+^ MDSCs, the suppressor function of Tregs, the IFN-γ production and tumor sizes. Analysis of these quantitative data were performed by One Way ANOVA for multiple groups or *t*-test for two groups. If One Way ANOVA showed statistical significance, then Pairwise Multiple Comparison Procedures (Holm-Sidak method) were done to compare various pairs of groups. Animal survival was analyzed with Log-Rank test. Statistical significance is assumed at p < 0.05. All statistical analysis was completed using the SigmaPlot 12.0 (Systat Software, Inc., San Jose, CA, RRID: *SCR_003210*).

## Results

### IL-15cx Shows Superior Bioactivity to Expand CD8 T Cells *In Vivo* in Comparison to an Equimolar Single Chain IL-15

First, we examined if IL-15 or IL-15cx we purchased could promote the expansion of CD8 T cells *ex vivo* and *in vivo*. Regardless of the presence of APCs either soluble IL-15 or IL-15cx alone in cell culture was insufficient to drive naïve CD8 T cells into cell-division cycle (**Supplementary Figure 2A**). However, IL-15 or IL-15cx in the media greatly prevented cell death of CD8 T cells. In contrast, almost all cells underwent cell death while there was IL-15Rα alone or in the absence of cytokines. A similar phenomenon was seen when IL-7 was added to the media instead of IL-15 (**Supplementary Figure 3**). Noticeably, the combination of IL-7 with IL-15 or IL-15cx was able to drive a small percentage of T cells into cell cycle resulting in a moderate proliferation with 3-4 cell divisions in 5 days. Nevertheless, after intraperitoneal administration of IL-15cx CD8 T cells from both spleens and lymph nodes showed a large expansion with at least 6-7 divisions of proliferation in 5 days (**Supplementary Figure 2B**). Contrastingly, the same molar amount of IL-15 was inefficient to promote CD8 T cell proliferation *in vivo*.

### The Absence of T-Bet Diminishes the Capacity of CD8 T Cell Proliferation Due to IL-15 or IL-15cx

**Figure 1:**
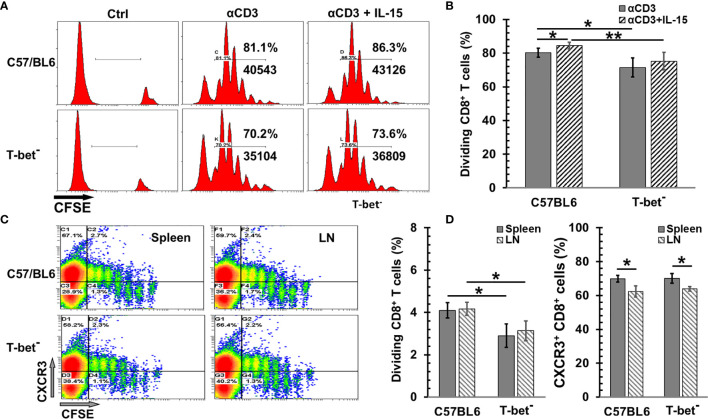
The impact of T-bet on CD8 T cell proliferation by interleukin 15 (IL-15) or IL-15cx. **(A, B)** T-bet influenced *ex vivo* CD8 T cell proliferation with or without IL-15. Unfractionated naïve CD8 T cells from C57BL/6 or T-bet^-^ mice were labeled with CFSE (5 µM) and then incubated with irradiated antigen presenting cells and αCD3 (0.5 *μ*g/ml) with or without IL-15 (1 ng/ml). Three days later, cells were analyzed by flow cytometry. **(A)**, Flow cytometric analysis of one representative experiment from four independent experiments. **(B)** Percentages of dividing (diluted CFSE) CD8 T cells in total gated live cells (n = 4). **(C**, **D)** T-bet was involved in *in vivo* CD8 T cell proliferation by IL-15cx. Unfractionated naïve CD8 T cells from C57BL/6 or T-bet^-^ mice were labeled with CFSE (5 µM) and then injected into tail veins of naïve C57BL/6 mice. Five days later, animals were euthanized, and tissue collection and analysis were carried out. **(C)** Flow cytometric analysis of one representative mouse from three mice. **(D)** Percentages of dividing (diluted CFSE) CD8 T cells in total gated live cells and Percentages of CXCR3^+^ cells in total CD8^+^ T cells (n = 3). Error bars represent standard deviations. *p < 0.05 and **p < 0.01 by One Way ANOVA.

T-bet plays a critical role in the CD8 T cell proliferation and differentiation by IL-12 (33), and the fate of effector and memory CD8 T cells (34, 35). However, the impact of T-bet on the IL-15-mediated naïve CD8 T cell proliferation is unclear. To investigate if T-bet participates in naïve CD8 T cell proliferation by IL-15, CD8 T cells isolated from T-bet^-^ mice were used to assess the bioactivity of IL-15 *ex vivo* and *in vivo*. Since IL-15 alone is inefficient for proliferation of CD8 T cells *ex vivo*, the precoated anti-CD3 antibody (αCD3) was added in cell culture. Both wild-type and T-bet^−^ CD8 T cells greatly responded to αCD3 initiated proliferation (**Figure 1A**). Lack of T-bet reduced T cell expansion by 11% (p = 0.029) (**Figure 1B**). The addition of IL-15 was able to further enhance the αCD3 elicited expansion by approximately 5% in both wild-type and T-bet^-^ CD8 T cells. However, lack of T-bet appeared to increase the variation of cell proliferation in response to αCD3 with or without IL-15 which led to no statistical significance in T-bet^-^ cells (p = 0.362) comparing to a significant change in wild-type T cells (p = 0.047). We then evaluated the impact of T-bet on the proliferation of CD8 T cells *in vivo* by IL-15cx. As shown in **Figures 1C, D**, T-bet^-^ CD8 T cells were able to expand but with a 30% reduction in cell frequency than wild-type CD8 T cells (p = 0.035). However, there was essentially no difference in the upregulation of CXCR3, an important chemokine receptor for T cell function, between wild-type and T-bet^-^ CD8 T cells. A small (9~11%) but significant decrease of CXCR3^+^ cells was nonetheless observed in CD8 T cells from lymph nodes in contrast to splenic CD8 T cells regardless of their T-bet status.

### IL-15 Antagonizes the Suppression of Tregs to Cytotoxic T Cell Proliferation and IFN-γ Release

**Figure 2:**
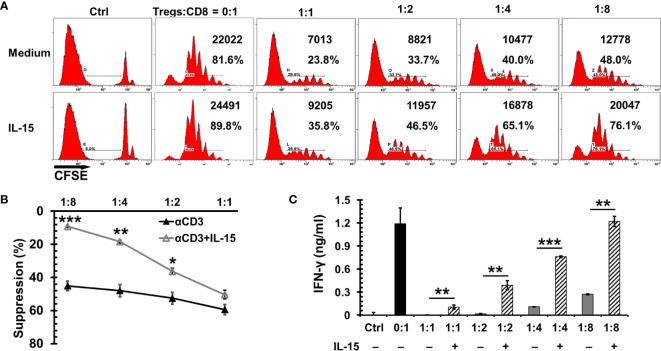
Interleukin 15 (IL-15) antagonized the suppression of Tregs to CD8 T cell proliferation and cytotoxic function. CD44^hi^CD8 T cells from C57BL/6 mice were labeled with CFSE (5 µM) and then incubated with irradiated antigen presenting cells, αCD3 (0.5 *μ*g/ml), and natural Tregs at various ratios (of Tregs vs CD8 T cells indicated in the Figure) with or without IL-15 (1 ng/ml). Three days later, cells were analyzed by flow cytometry. **(A)** Flow cytometric analysis of one representative experiment from four independent experiments. The absolute numbers and percentages of dividing CD8 T cells in total gated live cells were indicated. **(B)** IL-15 diminished the suppressor function of Tregs to CD8 T cell proliferation. The suppressor function of Tregs was normalized by CD8 T cell proliferation without the presence of Tregs (n = 4 independent experiments). **(C)** IL-15 promoted cytotoxic CD8 T cell function under the presence of Tregs. Supernatants collected from **(B)** were used to measure IFN-γ production by ELISA. Error bars represent standard errors **(B)** or standard deviations **(C)**. *p < 0.05, **p < 0.01 and ***p < 0.001 by *t*-test.

Tregs are present in the tumor microenvironment of breast cancer and are associated with unfavorable prognosis (36). Dr. Cerf-Bensussan’s group (37) reported that IL-15 impeded the suppressor function of human CD4^+^CD25^+^ Tregs to autologous CD4 or CD8 T cells. To examine if this is also true in mice, CD44^hi^ CD8^+^ T cells were isolated to carry out the Treg suppression assay. As seen in **Figure 2A**, natural Tregs showed a potent suppressor function to the proliferation of CD44^hi^CD8^+^ effector/memory-like T cells by αCD3 stimulation in a dose dependent manner. In the presence of IL-15, the suppressor function was reduced by 9.1%, 14.2%, 29.5%, and 35.9%, respectively with effector T cells versus (vs) Tregs at ratios 1:1, 2:1, 4:1, and 8:1 (**Figure 2B**). Of note, the reversal of IFN-γ release from memory-like T cells was more pronounced than the cell expansion (**Figure 2C**). The increase of IFN-γ production by IL-15 was correlated with the Tregs vs effector ratio as well. Without IL-15, IFN-γ release was severely suppressed by Tregs even at a high ratio of effector T cells versus Tregs, which indicates the cytotoxic function of memory CD8 T cells was impaired. At low ratios of effector T cells vs Tregs (2:1 or 1:1) there was no obvious IFN-γ release by CD8 T cells even when these cells exhibited visible proliferation. At an effector vs Treg 8:1 ratio, the IFN-γ production was decreased by 77% with a reduction of 45% cell proliferation. However, in the presence of IL-15 (1 ng/ml) the IFN-γ release was completely recovered to the level of memory cells without the suppression of Tregs. Noticeably, cell proliferation was still below the level of no Treg control. This phenomenon of IFN-γ release outpaced cell proliferation suggests IL-15 either impedes the suppressor function of Tregs or renders memory T cells more resistant to the Treg suppression.

### Intraperitoneal IL-15cx Results in Breast Cancer Growth Inhibition Associated With CTL Amplification and MDSC Reduction

**Figure 3:**
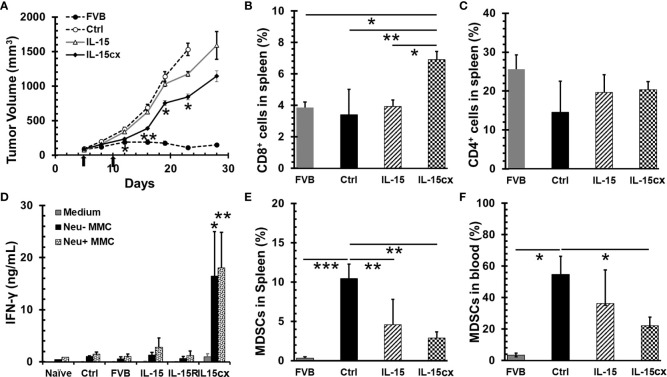
A moderate tumor growth inhibition by systemic administration of interleukin 15 (IL-15) was associated with the promotion of CD8 T cells and antitumor specific immunity and the reduction of myeloid-derived suppressor cells (MDSCs). Breast tumors were established by injections of 5 × 10^6^ neu^+^-MMC cells in the left posterior mammary fat pad of parent female FVB mice (FVB, n = 7) or Her2/neu transgenic FVBN202 mice. After tumor inoculation days 5 and 10 (indicated by arrows), tumor-bearing FVBN202 mice were treated with i.p. PBS as controls (Ctrl, n = 10), IL-15 2.5 µg (IL-15, n = 8), IL-15Rα-Fc 10 µg (IL-15R, n = 5), or equimolar IL-15cx (IL-15cx, n = 8). **(A)** Growth curves of neu^+^-MMC breast tumors. (B–F) Animals were euthanized at day 28. Blood and spleens were collected from three mice per group to examine immune cells or their function. **(B)** Percentages of CD8^+^ T cells in total gated live splenocytes. **(C)** Percentages of CD4^+^ T cells in total gated live splenocytes. **(D)** IFN-γ release from splenocytes cocultured with medium, irradiated neu^–^MMC or neu^+^-MMC for 24 h. No tumor-bearing FVBN202 mice were used as naïve mice control (Naive). **(E)** Percentages of CD11b^+^Gr-1^+^ MDSCs in total gated live splenocytes. **(F)** Percentages of CD11b^+^Gr-1^+^ MDSCs in total gated peripheral blood mononuclear cells (PBMCs). Error bars represent standard errors **(A)** or standard deviations **(B–F)**. *p < 0.05, **p < 0.01, and ***p < 0.001 by One Way ANOVA. p values between two groups were indicated **(B, C, E, F)**. If not indicated **(A, D)**, it means significant to all other groups.

To evaluate the therapeutic efficacy of IL-15cx for breast cancer, neu^+^-MMC tumors were initiated in Her2/Neu transgenic female FVBN202 mice or parent FVB mice. As seen in **Figure 3A**, neu^+^-MMC tumors fluctuated to grow in FVB mice in contrast to rapid growth in transgenic FVBN202 mice. Seven days after the first dose of IL-15cx treatment tumor growth was slower than that in control mice. This growth inhibition by IL-15cx therapy was maintained till the endpoint or euthanasia of animals. Nevertheless, no complete tumor regression was observed among the 8 mice treated with IL-15cx. On the other hand, IL-15 (**Figure 3A**) or IL-15Rα-Fc (Data not shown) treatment did not show any significant tumor growth inhibition. Animals were euthanized at the endpoint, and blood and spleens were collected to examine changes in CD11b^+^Gr1^+^ MDSCs, T cells and IFN-γ release after re-stimulation with tumor antigens. CD8^+^ T cells in spleen were significantly higher in the IL-15cx treated tumor mice than in control or the IL-15 treated tumor mice (**Figure 3B**). Although CD4^+^ T cells increased approximately 30% in both IL-15 and IL-15cx treated mice compared to control tumor mice, the difference was not statistically significant (**Figure 3C**). There was a dramatic 6 ~ 18-fold increase of IFN-γ release from the IL-15cx treated splenocytes incubated with irradiated neu^+^ or neu^-^ -MMC cells in comparison with control, FVB, IL-15, or IL-15Rα treated splenocytes (**Figure 3D**).

The increase of MDSCs is associated with tumor burden in breast cancer patients (38) and mouse mammary cancer models (39, 40). Consistent with literature reports, MDSCs were remarkably escalated in both blood and spleen of tumor mice (**Figures 3E, F**). The treatment with IL-15cx was able to greatly decrease the frequency of MDSCs in both blood and spleen whereas IL-15 treatment significantly reduced MDSCs in the spleen but had a less pronounced impact on these cells in the blood. Of note, the frequencies of MDSCs in blood or spleen in IL-15 treated animals were still six- to eight-fold higher than those in FVB mice.

### The Depletion of MDSCs Has No Effect On Mammary Cancer Growth and Therapeutic Efficacy of IL-15cx

**Figure 4:**
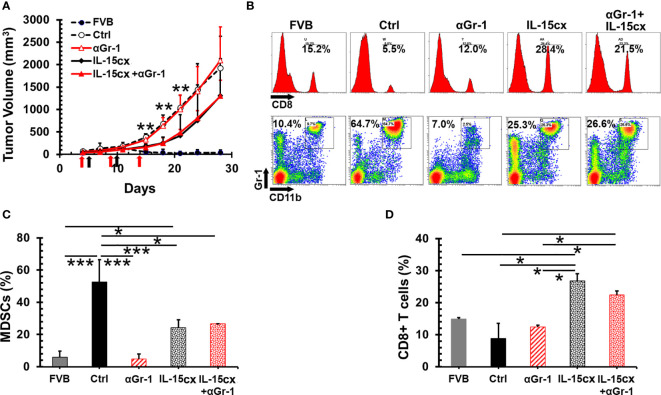
No therapeutic effect of the myeloid-derived suppressor cell (MDSC) depletion on mammary tumor growth and IL-15cx treatment. Breast tumors were established by injections of 2.5 × 10^6^ neu^+^-MMC cells in the left posterior mammary fat pad of parent female FVB mice (FVB, n = 4) or Her2/neu transgenic FVBN202 mice. Tumor-bearing FVBN202 mice were treated with i.p. PBS as controls (Ctrl, n = 4), anti-Gr-1 Ab 100 µg (αGr-1, n = 4), IL-15cx 12.5 µg (IL-15cx, n = 4), or both anti-Gr-1 Ab and IL-15cx (IL-15cx+αGr-1) (n = 4). Anti-Gr-1 Ab was given i.p. at days 4, 9, and 14 (indicated by red arrows) while IL-15cx was administrated i.p. at days 5 and 10 (indicated by black arrows). **(A)** Growth curves of neu^+^-MMC breast tumors. (B–D) Blood was collected *via* tail vein bleeding from three mice per group to examine immune cells. p values mean Ctrl or αGr-1 vs IL-15cx or IL-15cx+ αGr-1. **(B)** Flow cytometric analysis of one representative mouse from three mice. The percentages of CD8^+^ T cells or CD11b^+^Gr-1^+^ MDSCs in total gated live peripheral blood mononuclear cells (PBMCs) were indicated. **(C)** Percentages of CD11b^+^Gr-1^+^ MDSCs in total gated live PBMCs. **(D)** Percentages of CD8^+^ cells in total gated live PBMCs. Error bars represent standard deviations. *p < 0.05, **p<0.01, and ***p < 0.001 by one-way ANOVA.

An overwhelming number of MDSCs are present in mice with tumors, and the levels of these suppressor cells are still much higher in tumor bearing transgenic mice treated IL-15 or IL-15cx than parent FVB mice (**Figures 3E, F**). This made us question whether further removal of excessive MDSCs could slow tumor growth and enhance the antitumor activity of IL-15cx. Surprisingly, the depletion of MDSCs with anti-Gr-1 antibody had no impact on the tumor growth in control mice (**Figure 4A**) although MDSCs in blood were brought down from 53% in control mice to the same level (5%) in FVB mice (**Figures 4B, C**). And interestingly, the administration of IL-15cx made MDSCs resistant to the cytotoxicity of anti-Gr-1 antibody. The frequency (~26%) of MDSCs in the blood of mice treated with both IL-15cx and anti-Gr-1 antibody maintained the same level (~24%) in animals treated with IL-15cx alone. Regardless of the MDSC depletion status, anti-Gr-1 antibody failed to increase CD8 T cells in both control and IL-15cx treated animals (**Figures 4B, D**).

Unfortunately, no complete tumor regression was achieved in mice treated with IL-15, IL-15cx with or without the depletion of MDSCs. All mice were euthanized due to primary tumor growth having reached the endpoints in the approved IACUC protocol. Therefore, long-term antitumor immunity could not be assessed for the systemic treatment with IL-15 or IL-15cx.

### Intratumoral GET With Plasmid IL-15/IL-15Rα Results in a Survival Benefit for Mammary Carcinoma Bearing Mice Which Is Associated With an Early Increase of Local Cytotoxic Cells and Long-Term Memory T Cells Resulting From Complete Tumor Regression

**Figure 5:**
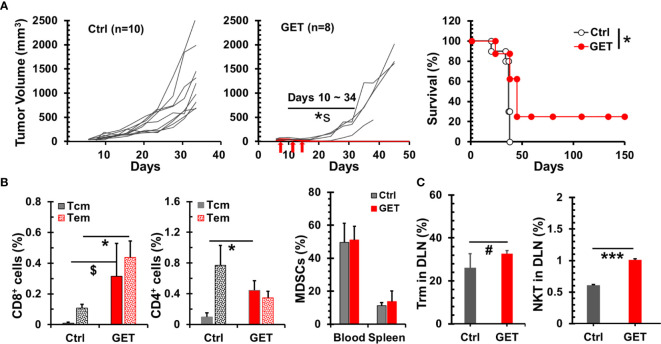
Therapeutic effect of intratumoral gene electrotransfer (GET) with plasmid interleukin 15 (IL-15)/IL-15Rα in breast tumor. Breast tumors were established by injections of 1 × 10^6^ 4T1-luc cells in the left posterior mammary fat pad of female Balb/c mice. Tumors (30–80 mm^3^) were treated with intratumoral saline (Ctrl, n = 10) or plasmid DNA IL-15/IL-15Rα (GET, n = 8) with electrical pulse deliveries at days 0, 4, and 7 (indicated by arrows). **(A)** Growth curves of 4T1-luc breast tumors and survival curves of tumor bearing animals. *^s^ p = 0.008–0.038 between Ctrl and GET groups during days 10–24 by *t*-test. *p < 0.05 between Ctrl and GET treatment by Log-Rank test. **(B)** Percentages of memory CD8/CD4 T cells in PBMCs. Flow cytometric analysis was performed at day 15 for tumor mice (Ctrl, n = 3) or day 107 for long-term tumor free mice after GET treatment (GET, n = 2). **(C)** Systemic and regional changes of immune cells after GET. Two days after the completion of GET treatment, animals (n = 5) were euthanized to analyze MDSCs in blood and spleens, and CD103^+^CD8^+^ tissue resident memory cells and Nkp46^+^CD3^+^ NKT cells in draining lymph nodes. Error bars represent standard deviations. ^$^p = 0.074, ^#^p = 0.058, *p < 0.05 and ***p < 0.001 by *t*-test.

The delivery of plasmid IL-15 by GET into tumor tissue led to the regression of B16 melanoma in mice (41), hence we investigated if this strategy could benefit breast cancer bearing animals. GET with plasmid IL-15/IL-15Rα did result in 4T1-luc tumor growth inhibition or regression (p = 0.008–0.038 between Ctrl and GET groups during days 10–24 in **Figure 5A**). Importantly, 25% (2/8) of animals obtained complete tumor regression and remained tumor free following GET with plasmid IL-15/IL-15Rα. Noticeably, blood drawn from these two mice that were tumor free over 3 months exhibited an increase of memory T cells including effector/central memory CD8^+^ and central memory CD4^+^ T cells compared to control tumor mice (**Figure 5B**). To explore what types of cells likely responded to intratumor GET treatment, 2 days after the completion of GET treatment, animals were euthanized to analyze immune cells in draining lymph nodes, blood and spleen. GET treatment had no impact on MDSCs (**Figure 5C**), Tregs and NK cells (data not shown) in blood and spleen but did increase CD8^+^CD103^+^ tissue resident T cells by 25% (p = 0.058) and CD3^+^NKp46^+^ NKT cells by 66% (p < 0.001) (**Figure 5C**) in draining lymph nodes. There was a heterogeneous but insignificant change of T cells in spleen and blood (**Supplementary Figure 4**). While one of five treated mice showed a threefold increase in T cells in the spleen and two mice exhibited a 40-60% increase, there was no apparent increase in the other two GET treated mice. These preliminary data suggest a distinct mechanism of intratumoral GET that initially promotes local antitumor immunity versus systemic enhancement of T cell proliferation and function by IL-15cx.

The two mice treated with GET and were long-term tumor free were challenged with live 4T1-luc cells. Both animals rejected breast tumor growth. This result is in line with increases of memory T cells (**Figure 5B**) further suggests long-lasting immunity can be induced by intratumoral GET treatment.

## Discussion

IL-15 as a monotherapy for solid tumors is not as effective as expected (42). Two major strategies including IL-15 superagonist and combination therapy have been pursued to improve its therapeutic efficacy. A number of studies have demonstrated the advantages of IL-15 superagonist over free monomeric IL-15, such as a longer half-life (20, 23, 43), better *in vivo* expansion of cytotoxic T/NK cells, and higher antitumor activity (22–24). We report here preformed IL-15cx can significantly expand CD8 T cells *in vivo*. T-bet apparently plays a certain, but not major, role in IL-15 driving T cell proliferation whereas CXCR3 upregulation by IL-15cx is independent of T-bet. Two doses of systemic IL-15cx showed a moderate antitumor activity for mouse breast cancer, which is likely associated with the promotion of cytotoxic T cell expansion and function enhancement. However, a successful depletion of MDSCs does not convert to any therapeutic benefits. We have also found IL-15cx antagonizes the depletion of MDSCs. Importantly, a significant antitumor activity including long-term complete tumor regression has been shown in animals treated with intratumoral delivery of IL-15/IL-15Rα co-expressing DNA. Further analysis indicates that the promotion of regional cytotoxic T cells is potentially involved in antitumor immunity.

IL-15 alone is able to induce a strong amplification of CD44^hi^ memory phenotype CD8 T cells both *in vitro* and *in vivo* but hardly stimulates the proliferation of CD44^low^ naïve CD8^+^ T cells (44). However, when examining unfractionated CD8 T cells in our study, few cells were found to be driven by IL-15 into one-to-two cell divisions then halted, both *in vitro* and *in vivo*. Our *in vitro* results can be explained when a very low concentration of IL-15 [here 1 ~ 10 ng/ml vs 10 ~ 1,000 ng/ml (44)] is used. Though comparable *in vivo* doses of IL-15 [here 2.5 µg vs 1 ~ 4 µg/mouse (44)] were adopted in both studies, a significant portion of CD8 T cells were found proliferating as indicated by BrdU positive cells in Dr. Zhang’s report (44). Nevertheless, other groups (20, 23) reported inefficient proliferation with a comparable dose of IL-15 when a CFSE labeling approach was employed. Therefore, the disparity is likely due to two distinct methods; otherwise, it suggests BrdU incorporation is more sensitive than CFSE labeling at measuring cell stimulation.

The JAK1/3 and STAT3/5 signaling pathways are utilized by IL-15 for T cell activation (8, 45), and T-bet is a known essential regulator of T cell differentiation and function (46). Although T-bet has been demonstrated as a critical factor for antigen-specific CD8 T cell function development (33), according to Dr. Glimcher’s report, it plays an insignificant role in cell proliferation from antigen-driven activation or αCD3/CD28 stimulation (47). However, our data showed that T-bet is partially involved in αCD3-induced *ex vivo* (**Figures 1A, B**) and IL-15cx-initiated *in vivo* (**Figures 1C, D**) naïve CD8 T cell proliferation. Noticeably, T-bet^-^ and wild-type OT-I TCR CD8 T cells were examined in above mentioned Dr. Glimcher’s study. The different source of CD8 T cells could explain this discordance which can be validated by well-designed experiments. Undoubtedly, TCR engagement or αCD3 signaling is a major driver of naïve CD8 T cell stimulation; whereas, IL-15 signaling appears more important in the generation and reactivation of memory-like CD8 T cells (44, 48–50). We have noticed that T-bet has a greater impact on the CD8 T cell proliferation *in vivo* than *ex vivo* due to IL-15 or IL-15cx. T-bet is a key regulator for Th1 cytokines (IL-12/IFN-γ) which promote CD8 T cell proliferation. Therefore, the deficiency of T-bet likely diminishes the response of CD8 T cells to Th1 cytokines *in vivo* in lymphoid tissues, including spleen and lymph nodes.

Both CD8 T cells and NK cells have been deemed necessary for IL-15cx or the superagonist to mediate antitumor immunity (24). However, in some circumstances, if NK1.1 cells become a major responder, the long term immunity or immune memory cannot be established (51). The importance of CD8 T cells has also been elaborated by Dr. Knudson’s group whereby the tumor-resident, rather than newly infiltrating CD8 T cells, were found to be responsible for the destruction of mouse melanoma (22). Our study is consistent with these literature findings. Partial tumor regression is only seen in mice treated with IL-15cx with a significant increase of CD8 T cells. On the other hand, no antitumor activity was observed in animals treated with IL-15 (**Figure 3**) or anti-Gr-1 alone, whereby the findings are associated with a depletion of MDSCs and no increase in CD8 T cells (**Figure 4**). In addition to expansion of existing antigen specific CD8 T cells, our *ex vivo* functional assays of Tregs suggest IL-15cx likely enhances the function of CD8 T cells as well (**Figure 2**). Interestingly, a dramatic release of IFN-γ from splenocytes in response to tumor antigens is discovered in IL-15cx treated mice but not in parental FVB mice with spontaneous rejection of neu^+^-MMC (**Figure 3**). This finding suggests a different antitumor mechanism involved between transgenic or non-neu transgenic FVB mice. The fact that splenocytes responded to both neu^+^ and neu^–^MMC indicates antitumor immunity is not dependent on HER2, oncoprotein but rather on other tumor specific or associated antigens.

Despite very potent bioactivity of IL-15 or IL-15cx at promoting cytotoxic T/NK cells, IL-15 immunotherapy appears ineffective at destroying poorly immunogenic primary tumors such as breast cancer. Both Drs. Kim (24) and Knudson (25) reported that an IL-15 superagonist ALT-803 had no impact on primary 4T1 breast tumor but could prolong animal survival if the primary tumor was removed by surgery. The survival benefit comes from the reduction/elimination of disseminated tumor cells or antimetastatic effect. Ineffective eradication of primary tumor is likely because of (1) immune suppressive tumor microenvironment of 4T1 breast tumor (31), (2) T cell exhaustion (51) and (3) lack of tumor resident memory T cells (22). Here we have examined a different strategy of IL-15 immunotherapy, intratumoral GET, in a 4T1 breast cancer model. Though there is no notable and uniform increase/decrease of circulating T, NK cells, Tregs or MDSCs, a significant tumor regression and survival benefit has been achieved while regional cytotoxic cells greatly rise (**Figure 5**). Our discovery, together with above mentioned reports, emphasize the importance of local immunotherapy with IL-15 or IL-15cx to elicit effective antitumor immunity for a poorly immunogenic tumor or “cold” tumor.

Combination immunotherapy has been proposed to enhance the efficacy of IL-15 (42). MDSCs are predominant in 4T1 breast tumor and have been reported to associate with tumor burden or late stage breast tumor (38). MDSCs have been suggested as a target for the enhancement of immunotherapy and shown an improved immunity of cancer vaccine in mouse and human lung cancer (52, 53). However, our data appears to disagree with this approach for the 4T1 model and IL-15 immunotherapy. The depletion of MDSCs does not change the growth pattern of 4T1 tumor. It is probably other immune suppressive cells such as Tregs and tumor associated macrophages taking over or compensating for the immunosuppression of MDSCs. We have found that the depleting antibody loses its efficacy when IL-15cx is present (**Figure 4**). This result is consistent with the promotion of CD11b^+^ myeloid cells by IL-15 (54, 55). However, in our study IL-15 or IL-15cx treatment has been shown to decrease MDSCs in tumor mice (**Figure 4**). Taken together, the impact of IL-15cx on MDSCs is complicated and needs further investigation to understand its contribution to the antitumor activity.

We would like to point out there are some limitations of this research. Metastatic diseases including organ metastasis and circulating tumor cells are not evaluated. It is possible that systemic or local delivery of IL-15/IL-15cx could reduce metastatic burdens as reported by Drs. Kim and Knudson (24, 25), in which primary tumor should be removed after the treatment has been done. In terms of mechanisms of IL-15cx therapy, CD8 T cells are emphasized in this study. Other cells, such as NK and NKT cells that can respond to IL-15cx as well, should be investigated to gain a better picture of immune response. Our limited data suggest that increases of regional tissue resident memory T and NKT cells are associated with effectiveness of IL-15cx therapy. Further analysis of dynamic changes of immune cells in tumor microenvironment should help us understand the different mechanisms in terms of local vs systemic IL-15cx treatment or effective vs ineffective IL-15cx treatment. Our future research is to enhance the efficacy of IL-15cx treatment and elucidate the mechanisms of heterogeneous immune outcomes.

In conclusion, our study demonstrated the therapeutic potential of IL-15cx as a potential breast cancer immunotherapy. We discovered that T-bet played a certain role in IL-15cx driving CD8 T cell proliferation. Systemic administration of IL-15cx resulted in a moderate antitumor activity in the mouse breast tumor model though a remarkable increase of CD8 T cell expansion and antitumor cytotoxic function was observed. Notably, intratumoral GET with plasmid IL-15/IL-15Rα exhibited a significant tumor regression and survival benefit even though significant changes of systemic cytotoxic cells and immunosuppressor cells were not observed initially. It appears early increases of regional cytotoxic cells were critical to the antitumor activity of GET treatment. In addition, we demonstrated the depletion of MDSCs was ineffective to treat mouse breast cancer. Therefore, our research proposed local delivery with IL-15cx should be further optimized and considered for breast cancer immunotherapy. More effective IL-15cx based combination therapies should be explored as well.

## Data Availability Statement

The original contributions presented in the study are included in the article/**Supplementary Material**. Further inquiries can be directed to the corresponding author.

## Ethics Statement

The animal study was reviewed and approved by The Institutional Animal Care and Use Committee (IACUC) at Virginia Commonwealth University and the IACUC at Old Dominion University.

## Author Contributions

SG and RS conceived of the study. SG, RS, AN, and RH conducted the experiments. SG analyzed and interpreted the data. SG wrote the manuscript. All authors contributed to the article and approved the submitted version.

## Funding

This work was supported by funding from the National Cancer Institute grant R21 CA229939 to SG and funding from the Thomas F. and Kate Miller Jeffress Memorial Trust to RS.

## Conflict of Interest

The authors declare that the research was conducted in the absence of any commercial or financial relationships that could be construed as a potential conflict of interest.
