# Supplementary material for: IL-15/IL-15Rα Heterodimeric Complex as Cancer Immunotherapy in Murine Breast Cancer Models
Source: Front Immunol. 2021 Feb 8;11:614667. doi: 10.3389/fimmu.2020.614667 (PMC7897681; doi:10.3389/fimmu.2020.614667)
Supplement: Supplementary file 1 [file DataSheet_1.docx]

**Supplement Materials for:**

**IL-15/IL-15Rα heterodimeric complex as cancer immunotherapy in murine breast cancer models**

Siqi Guo^1, 2^*, Ronald Smeltz^2^, Anthony Nanajian^1^, Richard Heller^1^

^1^ Frank Reidy Research Center for Bioelectrics, Old Dominion University. Norfolk, USA

^2^ Department of Microbiology & Immunology, Virginian Commonwealth University, Richmond, USA

*Correspondence to: Siqi Guo, email: [s2guo@odu.edu](mailto:s2guo@odu.edu)

**Suppl. Figures**

Suppl. Figure 1


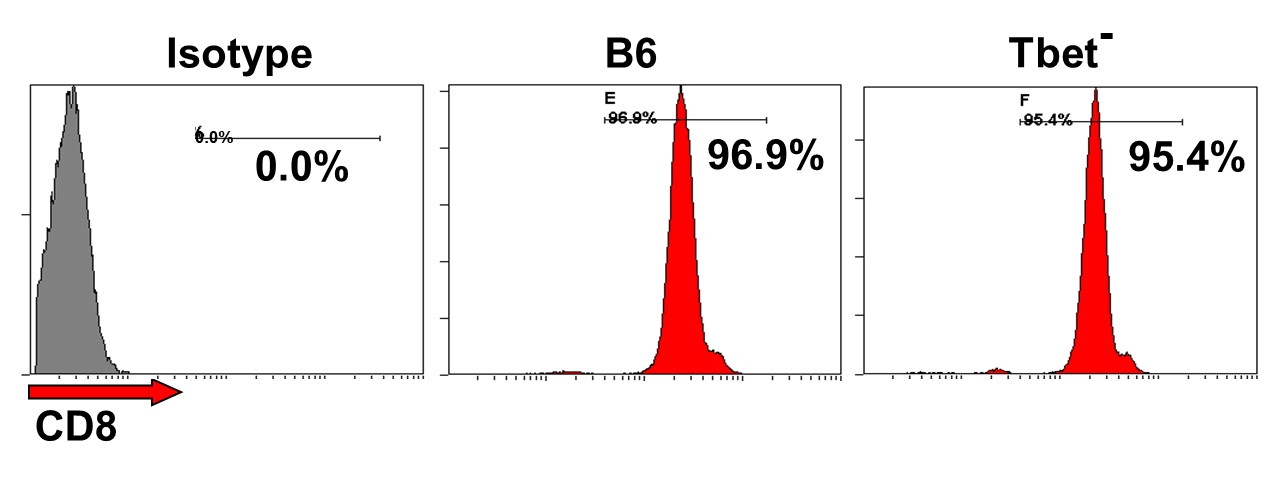


**Suppl. Figure 1. The purity of isolated CD8 T cells.** Flow cytometric analysis was performed to determine the purity of CD8 T cells isolated from C57BL/6 (B6) or T-bet^-^ mice (T-bet^-^). A typic result of T cell purity was showed.

Suppl. Figure 2


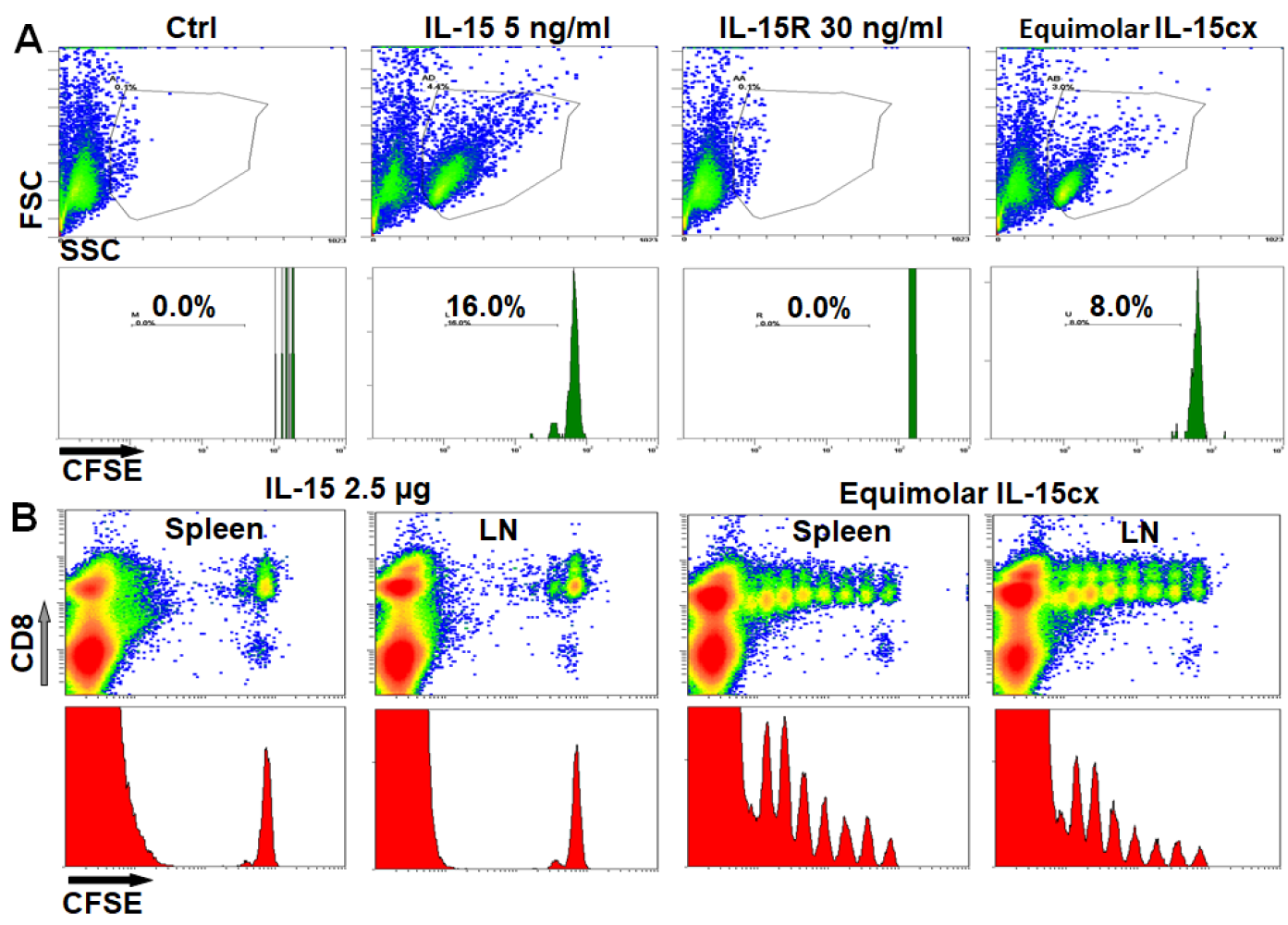


**Suppl. Figure 2. The bioactivity of IL-15 or IL-15cx to CD8 T cells.** Unfractionated naïve CD8 T cells from C57BL/6 mice were labeled with CFSE (5 µM) and then either cultured *ex vivo* (A) or injected into tail vein of naïve mice (B). (**A**) *Ex viv*o bioactivity of IL-15 or IL-15cx to CD8 T cells was analyzed by flow cytometry after 5 days of incubation. Results of one representative experiment from three independent experiments were shown here. (**B**) *In vivo* bioactivity of IL-15 or IL-15cx to CD8 T cells. Results of one representative mouse from two individual mice were shown here. The concentration of IL-15, IL-15Rα or IL-15cx in cell culture media (A) or the dose of cytokine per mouse (B) was indicated, respectively.

Suppl. Figure 3


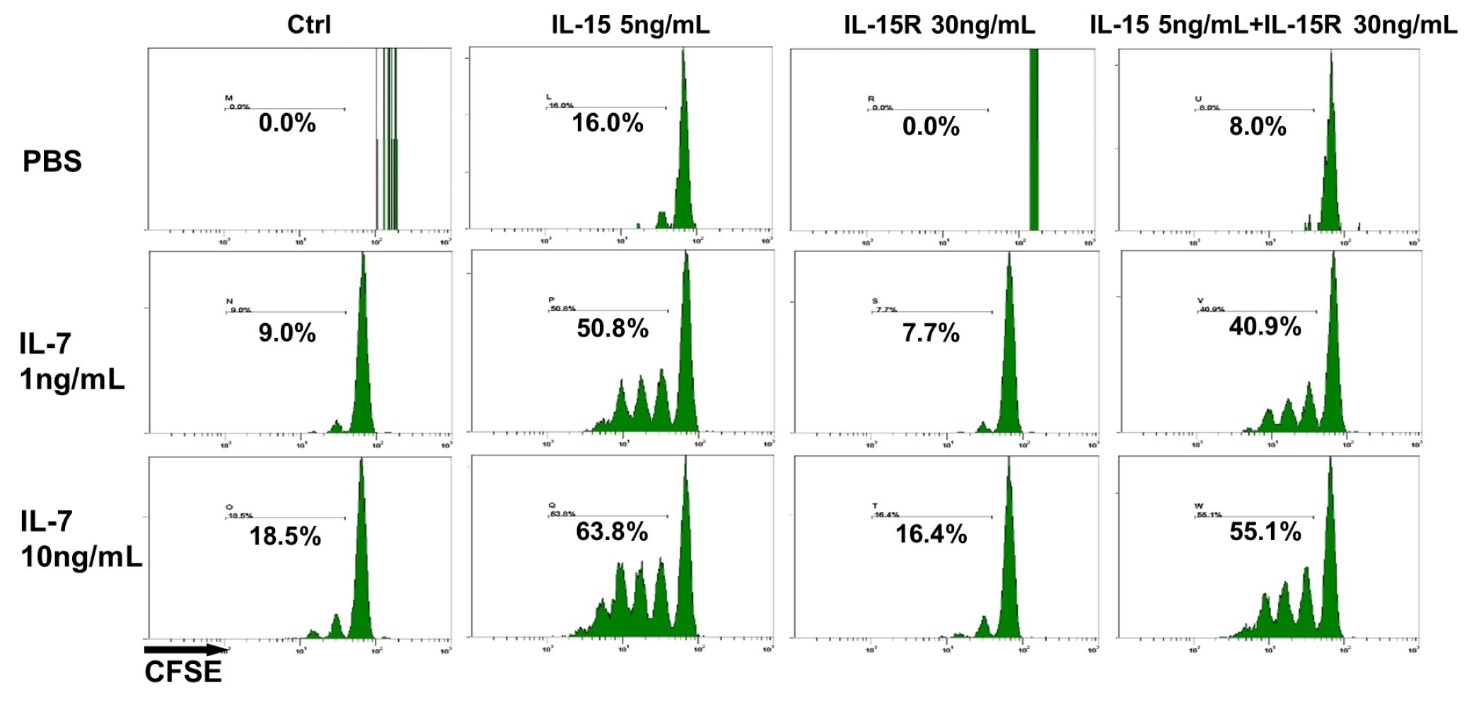


**Suppl. Figure 3. The bioactivity of IL-15, IL-7 or IL-15cx to CD8 T cells.** Unfractionated naïve CD8 T cells from C57BL/6 mice were labeled with CFSE (5 µM) and then cultured with various cytokines *ex vivo*. The bioactivity of IL-15, IL-7 or IL-15cx to CD8 T cells was analyzed by flow cytometry after 5 days of incubation. The concentration of IL-15, IL-7, IL-15Rα or IL-15cx in cell culture media and percentages of dividing (diluted CFSE) CD8 T cells in total gated live cells were indicated, respectively.

Suppl. Figure 4


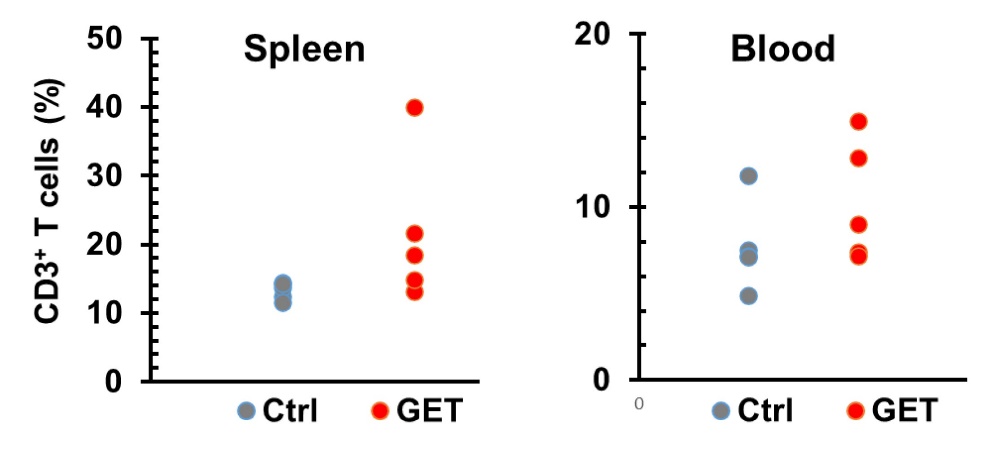


**Suppl. Figure 4. Changes of T cells following intratumoral GET with plasmid IL-15/IL-15Rα.** Breast tumors were established by injections of 1 × 10^6^ 4T1-luc cells in the left posterior mammary fat pad of female Balb/c mice. Tumors (30-80 mm^3^) were treated with intratumoral saline (Ctrl) or plasmid DNA IL-15/IL-15Rα (GET) with electrical pulse deliveries at days 0, 4 and 7. Two days after the completion of GET treatment, animals (n=5) were euthanized. Spleens and blood were harvested for the analysis of T cells by flow cytometry. Percentages of CD3^+^ T cells in spleen (**A**) or blood (**B**) were shown here.
